# Sensor-based measurement of fear avoidance and movement patterns of healthy individuals with mechanically induced lower back pain during movement tasks: Study protocol for an experimental non-interventional study

**DOI:** 10.1371/journal.pone.0358129

**Published:** 2026-09-17

**Authors:** Annika Heuler, Isabella Hebel, Robert Richer, Nicolas Rohleder, Björn M. Eskofier, Annika Genenger, Antonia Barke, Cornelia Weise

**Affiliations:** 1 Department of Psychology, Friedrich-Alexander-Universität Erlangen-Nürnberg, Erlangen, Germany; 2 Department Artificial Intelligence in Biomedical Engineering, Friedrich-Alexander-Universität Erlangen-Nürnberg, Erlangen, Germany; 3 Institute of Psychology, Universität Duisburg-Essen, Essen, Germany; University of Marburg: Philipps-Universitat Marburg, GERMANY

## Abstract

**Background:**

Chronic primary low back pain (CPLBP) is highly prevalent and associated with physical impairment and psychological distress. Fear avoidance (FA) is one contributing factor in the development and persistence of CPLBP. Previous studies proved existing connections between pain, physical activity, stress, and FA, which is also present in pain-free individuals. However, the roles of FA and psychophysiological stress responses have not been systematically disentangled and related to movement patterns combined in one standardized experiment involving pain induction.

**Methods:**

To address this gap, 76 pain-free participants (20: phase 1; 56: phase 2) will perform standardized movement tasks (Back Performance Scale, Sit-To-Stand-Test) with and without wearing a back pain simulator in a within-subject design. Movement will be recorded with wearable sensor-based and novel radar-based motion capture technology. Additionally, psychophysiological stress parameters (e.g., salivary cortisol, heart rate variability), and self-reports on stress, pain, and FA will be assessed. This will be followed by a qualitative feasibility and acceptability interview in phase 1. In this phase, we will evaluate the feasibility and acceptability of a novel back pain simulator enabling a standardized simulation of low back pain, and the motion capture technology. Phase 2 will investigate the effects of pain induction on self-reported pain, back pain-related movement parameters, and stress responses. Associations of these factors with FA will be analyzed. We anticipate high feasibility and acceptability. We expect higher pain ratings, lower mobility, and higher stress responses in the pain condition compared to the control condition. We expect FA to moderate mobility and stress responses in the pain condition.

**Discussion:**

This study protocol establishes an experimental paradigm to investigate the association of pain, FA, movement, and stress in healthy samples under controlled pain conditions. This paradigm aims to contribute to a deeper understanding of the interplay of psychological and physiological mechanisms in CPLBP.

**Trial registration:**

OSF, osf-registrations-rt2ca-v1. Registered April 2, 2025, https://doi.org/10.17605/OSF.IO/RT2CA; osf-registrations-vsmce-v1. Registered September 18, 2025, https://doi.org/10.17605/OSF.IO/VSMCE.

## Introduction

Chronic low back pain (CLBP) is a common health condition. Prevalences range from 3.9% and 25.4%, depending on factors such as age, sex, or the pain definition used [1]. According to the International Classification of Diseases (ICD-11), low back pain (LBP) is pain located between the inferior gluteal folds and the costal margin [2]. Pain that lasts longer than 3 months is considered chronic pain [3]. Two different types of chronic pain are distinguished in the ICD-11 [4]: chronic primary pain and chronic secondary pain. Chronic primary pain is characterized by significant distress and disability and not better accounted for by a specific secondary form of chronic pain. Regarding chronic low back pain, chronic secondary low back pain refers to consequences of a specific pathology (e.g., tumor, herniated intervertebral disc) or surgery [5]. Chronic primary low back pain (CPLBP) comprises all forms of chronic low back pain accompanied by distress or disability and not better accounted for by a chronic secondary pain; previously these were often – in felicitously – referred to as “non-specific”.

Biological, psychological, and social factors contribute to the development and persistence of CPLBP [6–8]. A central psychological factor in many CPLBP patients is the tendency to avoid physical activities due to the belief that such activities may worsen pain or cause (re-)injury [9]. This behavior, known as fear avoidance (FA; [10,11]) often relates to specific movements associated with back pain, such as lifting objects or bending down. FA is observed in up to 40% of CPLBP patients [12]. According to the original FA model, movement avoidance arises through classical conditioning: The affected person associates pain with specific physical activities, which are then perceived as threatening and potentially harmful as they might cause pain or further injury [9,13,14]. Neurobiological investigations failed to identify neural correlates of the hypothesized fear response [15,16], hypothesizing that beliefs and expectations are more relevant than a conditioned fear response. This is also supported by studies reporting FA beliefs in healthy, pain-free persons [17,18], who may have acquired them via semantic learning, e.g., due to the proliferation on back-related advice [19,20].

Irrespective of how FA beliefs arise, they can have wide-ranging consequences across physical, psychological, social, and societal domains [21]. On a physical level, continued inactivity may lead to deconditioning, increased pain sensitivity, and further functional decline, potentially resulting in a vicious cycle of disability [22,23]. Psychologically, FA is associated with feelings of helplessness [24], depressive symptoms [11,23], and general mental distress [23]. It has also been linked to poorer psychological and functional treatment outcomes in chronic pain patients [23]. Socially, FA can lead to disengagement from both professional and private life [21], leading to a reduced quality of life. These effects translate into both direct costs due to higher healthcare utilization and prolonged treatment and indirect costs, such as increased sick leave and work loss [25].

If avoidance of a movement is not possible, many CPLBP patients with FA exhibit guarded movements, such as stiffening to protect the spine [26,27]. These protective movements have been examined using surface electromyography (sEMGt), sensor-based motion capture systems, and psychometric assessments.

SEMGt studies have analyzed trunk muscle activity during gait tasks, with mixed results in relation to FA behavior [28,29]. Ghamkhar and Kahlee [29] reported increased activity in muscles associated with back pain, whereas Pakzad et al. [30] found no significant change in overall muscle activation levels, but observed altered variability in muscle activation patterns. Some studies employed sensor-based motion capture systems to assess FA-related movement patterns during potentially threatening movement tasks, both in persons with CPLBP [27] and pain-free individuals [26]. Matheve et al. [27] identified a reduced lumbar range of motion while Knechtle et al. [26] observed decreased lumbar flexion during tasks rated as potentially harmful. Most of these studies focus only on certain muscle groups, a single movement or mobility parameter, resulting in a lack of a standardized, integrated paradigm.

Together with fear-related behavioral patterns, stress responses are a relevant factor in CPLBP (e.g., [23]). Stress in this context refers to both physiological responses (i.e., activation of the hypothalamic-pituitary-adrenal (HPA) axis and the autonomic nervous system (ANS)) and psychological reactions to actual or anticipated threats. Stress can be triggered by pain itself, by movements, or by the expectation of pain. Patients with CPLBP often report elevated stress levels during or after physical activity [31] as well as anticipatory distress before movements perceived as harmful [32]. Like pain, stress is modulated by psychosocial factors such as fears, beliefs, and cognitive appraisals [33–36]. This suggests that individual differences in coping and interpretation of pain-related stimuli may influence stress responses, potentially interacting with FA mechanisms. Although altered stress regulation has been hypothesized in CPLBP, empirical evidence remains inconsistent [37], pointing to substantial heterogeneity in psychological and physiological stress responses. This emphasizes the importance of accounting for potential sources of variation in the interaction between stress and pain.

To integrate current findings and guide further research, Timmers et al. [37] proposed a threat learning model, including FA beliefs as a mediator between stress responses and chronic pain. Findings partially support and partially contradict this model [32,38]. The complexity of both acute stress responses (e.g., [35,39]) and chronic pain mechanisms [40] further complicates the understanding of their interaction [37]. As both stress and pain are complex and constantly interacting, research in FA, especially in relation with stress responses, can be challenging. We will approach these challenges with a standardized and integrated experimental paradigm considering different measurements for both FA and stress responses.

Our novel integrated paradigm will enable research on the interactions between FA, pain, actual movement patterns, and biopsychological stress responses, which has not been previously undertaken. Comprehensive research on FA faces methodological challenges, particularly with the integration of multimodal data, such as self-report data, movement information, and biopsychological stress markers. Most studies rely on self-report measures or imagery tasks, as actual movement tasks are burdensome, potentially painful for patients, and require complex equipment (e.g., [41]).

Advancements in sensor technology now allow simultaneous measurement of multiple parameters (e.g., movement kinematics, muscle activity), enabling more integrated assessments. Investigating FA in pain-free individuals offers additional insight, as guarded movements also occur in this group [26], though with notable differences. In contrast to pain-free individuals, patients show higher FA, lower functional capacity, and greater overgeneralization of fear [42,43]. Thus, data from actual movement behavior in pain conditions remain essential for understanding FA processes [27].

In summary, there is a lack of an experimental paradigm to investigate the association of pain, FA, movement, and stress under controlled pain conditions with focus on the integration of multimodal data. With the study described in this study protocol, we want to address this gap.

This study protocol comprises a study that is part of a subproject within the interdisciplinary Collaborative Research Center “Empatho-Kinaesthetic Sensor Technology (EmpkinS)”. The goal of EmpkinS is to research minimally invasive, patient-centered diagnostic approaches in medicine and psychology. Phase 1 serves as a proof of concept for both the measurement technologies and experimental procedures, paving the way for phase 2 and following studies. Phase 1 has two aims: (1) assessing the feasibility and acceptability of a back pain simulator to induce temporary lower back pain and (2) testing the feasibility and acceptability of the study setup, i.e., the pain-related movement tasks in combination with the employed sensor technology. The acceptability and feasibility of the back pain simulator and the study setup will be evaluated exploratory in a qualitative interview.

Phase 2 will examine differences between participants with and without experimentally induced lower back pain in back-pain-related movement parameters during standardized movement tasks and investigate the related stress responses. In addition, we will collect self-report data on FA and investigate whether FA moderates the movement parameters and stress responses in the pain condition.

The following hypotheses will be tested:

(1) The task-related ratings of pain intensity are higher in the condition with the back pain simulator (PainSim+) than without (PainSim-).
(2) The fear of movement ratings are higher in the condition with the low back pain simulator (PainSim+) than without (PainSim-).
(3a) The task-related ratings of motion impairment are higher in the condition with the back pain simulator (PainSim+) than without (PainSim-).
(3b) The mobility (measured through lumbar range of motion (LROM), lumbar flexion (LF), and movement velocity (MV)) is lower in the condition with the low back pain simulator (PainSim+) than without (PainSim-).
(3c) Observer ratings of performance of task execution are worse in the condition with the low back pain simulator (PainSim+) than without (PainSim-).
(4a) The task-related ratings of emotional distress are higher in the condition with the low back pain simulator (PainSim+) than without (PainSim-).
(4b) The subjective stress ratings is higher in the condition with the low back pain simulator (PainSim+) than without (PainSim-).
(4c) The heart rate variability (HRV) is lower in the condition with the low back pain simulator (PainSim+) than without (PainSim-).

In exploratory analyses, we will examine whether even in this analogue sample self-reported FA is associated negatively with mobility and HRV, and positively with subjective stress reports, salivary stress markers (alpha-amylase, cortisol), and pain ratings.

This integrated design allows for the combined assessment of physiological stress responses, behavioral changes, and movement-related aspects across standardized back-stressing movements.

## Materials and Methods

### Ethics approval and consent to participate

The study was approved by the Ethics Committee of the Medical Faculty of the Friedrich-Alexander-Universität Erlangen-Nürnberg (ethical approval codes for studies: 25–21-S) and will be conducted in accordance with the Declaration of Helsinki.

All participants will give written informed consent before testing. Prospective participants will be provided with information about the study and data protection when opening the screening-link. Additionally, the informed consent document will be available for the participants as a PDF via a download link prior to participation. If questions arise, participants can contact the study team at any time of study conduction via an email address noted on flyers, posters, etc. The informed consent consists of several parts: consent to participating in the study in general, use of salivary samples, data protection, video and audio recordings, and handling of incidental findings. This enables participants to agree to certain parts of the study procedures while not agreeing to others (e.g., salivary samples can only be used within the concerning study but not in studies with similar research questions).

### Study design

The study design is reported in line with the SPIRIT guidelines [44]. The experiment will take place in the EmpkinS laboratory in Erlangen, Germany. A repeated measures design will be used. Pain-free participants will execute standardized movement tasks with a mechanical low back-pain simulator (PainSim+) and without (PainSim-) a mechanical back-pain induction. An a priori computer-generated list based on random numbers created by one experimenter will be used to randomize the order of the conditions within each subject. S1 Fig provides an overview of the procedure of both phases.

### Eligibility Criteria

Participants are eligible for inclusion if they meet the following criteria:

1) Age between 18 and 65 years
2) Sufficient knowledge of German (native speaker or C2 level)
3) Body mass index (BMI) between ≥ 18 and ≤ 25 (due to the limited size range of the back pain simulator and Xsens suit, see below)
4) Body height ≥ 155 cm (setup requirement for back pain simulator and Xsens suit)
5) Good physical and mental health state (see exclusion criteria)

Exclusion criteria for participation are as follows:

- 1) Chronic back pain
- 2) Depression (exceed cut-off score for clinically relevant depression symptoms of 22 on the General Depression Scale (German: Allgemeine Depressionsskala; ADS-L; [45])
- 3) Serious diseases or conditions concerning the nervous system (e.g., severe epilepsy), musculoskeletal system (e.g., rheumatic arthritis), skin (e.g., acute neurodermatitis), and cardiopulmonary system (e.g., myocardial infarction); cancer, strokes; serious psychological disorders will be screened via the Web Screening Questionnaire for Common Mental Disorders (WSQ; [46])
- 4) Acute infectious diseases (e.g., HIV/AIDS)
- 5) Current pregnancy
- 6) Acute herniation (e.g., spinal disc herniation)
- 7) Acute back pain (ranging on a scale from 0 = *no pain* to 10 = *worst imaginable pain* between ≥ 5 and ≤ 10)
- 8) Acute muscle and/or skeletal pain interfering with daily activities
- 9) Diseases of the nerve roots and spine injuries
- 10) Osteoporosis of the spine or back
- 11) Surgery on or near the spine
- 12) Diabetes mellitus (back pain simulator requirement, inter alia because of associated skin and soft tissue changes and higher risk of skin infections; e.g., [47])

We will follow an adaptive design and may change eligibility criteria for phase 2 based on the phase 1 findings.

### Sample size calculation and recruitment

We aim to examine 76 participants (phase 1: 20, phase 2: 56). The sample size for phase 1 follows recommendations for feasibility pilot studies, as the purpose of phase 1 is to test procedures for phase 2 [48,49].

Sample size calculations for phase 2 were based on a repeated measures design and calculated with G*Power. Based on similar study setups [27], we anticipate a medium effect size of approximately *d* = .40 [50] resulting in a total sample size of *N* = 51 (parameters: *d* = .40, alpha = .05, 1 – beta = .80). To compensate for dropouts and possible technical issues leading to missing data (especially motion capture data) we will recruit 10% additional participants, resulting in a total sample of *N* = 56.

Recruitment for both phases is carried out and monitored by the study team. Phase 1 is currently being conducted (from June 2025 until the anticipated completion at the end of November 2025), phase 2 is planned to begin in January 2026 and continue for eight months. Each participant will be offered a compensation of €20. Alternatively, participating students can receive course credits.

### Procedure

In phase 1 we aim to recruit primarily students via social media, flyers, university mailing lists, and word-of-mouth recommendation. For phase 2, recruitment will be expanded to the general population using the same strategies, supplemented by postings on public university websites and advertisements in local and regional media (e.g., newspapers).

The procedure is identical in both phases, except for the follow-up interview, which is only part of phase 1 and any small adjustments to maximize acceptability that may result from it. For a detailed overview of the participant timeline and measures see S2 Fig. In phase 2, we will additionally use a second motion capture technology (for more details see description below) to assess movement patterns. Each phase includes an online questionnaire for eligibility screening and baseline data and an on-site lab appointment. During the lab-session, participants complete two rounds of standardized movement tasks, once with and once without the back pain simulator. Throughout the session, stress levels (via saliva samples, cardiac measures, and self-report), self-reported pain, movement data (motion capture suit), and for female participants salivary progesterone and estradiol will be assessed. In phase 1, an acceptability and feasibility interview follows the lab session.

### Online Questionnaire

Prospective participants can register online (via link or QR code) and complete a questionnaire containing the consent to the screening questionnaire, demographic variables, presence of diseases potentially influencing outcomes (e.g., hypothyroidism), current medication intake (e.g., hormone preparations, psychotropic drugs), and for women questions regarding menstrual cycle and hormonal contraceptives, and the screening for inclusion and exclusion criteria. Demographic variables include age, sex, gender, occupational group, highest educational qualification, and ethnic affiliation. If participants are not eligible, they are informed about exclusion. If eligibility criteria are met, participants are asked to complete questionnaires assessing fear avoidance (Tampa Scale for Kinesiophobia for general population; [51]), fear avoidance response (Avoidance-Endurance Questionnaire; [52]), pain catastrophizing (Pain Catastrophizing Scale; [53]), coping with pain and pain-related distress (Fragebogen zur Emotionalen Schmerzverarbeitung; English: pain processing questionnaire; [54,55]), and stress perception (Perceived Stress Scale; [56]). After completing the questionnaire, potentially eligible participants are contacted via e-mail or phone, provided with information about informed consent, and offered a lab appointment on a business day between 11 am and 8 pm. In case of non-response, participants will be reminded once via email or phone and otherwise excluded.

### Lab appointment

Prior to the lab appointment participants will be informed via e-mail about the following instructions and restrictions: Waking up at least three hours before the appointment, no eating and caffein consumption one hour before the appointment, no smoking or exercise two hours before the appointment, and wearing comfortable clothes with thin fabric (no dresses or skirts). The lab appointment begins with the experimenters obtaining written informed consent. Participants then provide a baseline saliva sample for stress assessment (s0), which includes carefully moving a cotton roll (Salivette®, Sarstedt, Nümbrecht, Germany) in their mouth for 1.5 min. Afterwards, their body weight, height, and waist and hip circumference are measured. Female participants additionally provide a saliva sample of 5 ml via passive drooling (pooling saliva in the mouth and allowing it to drip into the sample tube) for the analysis of progesterone and estradiol levels as they might influence pain perception [57,58] and stress responses [35,59]. The core part of the experiment is conducted under two conditions in randomized order, namely, once with and once without the back pain simulator to allow for within-subject comparison. Before each condition, participants complete the Short Stress State questionnaire (SSSQ-G; [60]) and provide another saliva sample (s1). Participants then perform six movement tasks: five from the Back Performance Scale [61] and a sit-to-stand task (comparable to [62]; adjusted arm position: hands are placed relaxed on the legs). Movement quality is rated by an experimenter (see Additional File 1), and a video of the movement execution is recorded. Throughout the tasks, participants wear an inertial measurement unit (IMU)-based motion capture suit (Xsens MVN, Movella, Henderson USA; for more details see [63]). In phase 2, this will be supplemented by the EmpkinS holographic 6D wireless locating technique and integrated localizable electromyography radio transponders (EmpkinS sensor technology; [64,65]). After each task, participants rate pain intensity, emotional distress, and pain-related impairment on a visual analogue scale (VAS). Following task completion, the SSSQ-G and a third saliva sample (s2) are collected. The entire procedure is repeated under the alternate condition (with/ without low back pain simulator), including an SSSQ-G assessment before and after the six movement tasks, as well as another saliva sample (s3) after the movement tasks. In total, each participant completes 2x6 movement tasks, provides 4 saliva samples, and 2x2 SSSQ-G assessments.

At the end of the lab session in phase 1, participants are interviewed about the experimental procedure to obtain qualitative feedback on the quality of the simulated back pain and the overall acceptance of the back pain simulator and the motion capture suit (see Additional File 2). The experiment will be conducted by two trained experimenters. Standardization will be ensured throughout the procedure. To maintain protocol adherence, experimenters follow a detailed checklist during the lab session, which also includes comment sections for any deviations from the protocol. If participants abort during the study, they will be asked about the reason, and their data will be excluded from analyses.

### Experimental procedure

#### Movement tasks (Back Performance Scale and Sit to Stand Task).

The Back Performance Scale (BPS) was developed to measure performance of mobility-related activity in back pain [61]. It comprises five tasks: the sock test, roll-up test, finger-floor, distance test, pick-up test, and lifting test (see Additional File 1). Performance regarding the participant’s effort and execution of the tasks is assessed by using a 4-point ordinal scale from 0 (best performance, e.g., “Can do the task with ease in varied ways”) to 3 (worst performance, e.g., “Cannot perform the task at all or need external support”) with individual wordings for scoring categories for each movement task [61]. In the lifting task, an additional specification is to put a time limit of one minute and count the repetitions of the participant. To ensure proper execution and not ambition, participants are not informed about this time limit beforehand. The whole BPS sum score ranges from 0 to 15 with lower scores indicating better performance and back mobility.

The sit-to-stand task (STST) was originally developed to measure performance of lower extremity muscles [66] and has also been used in the context of CBLBP [67]. The task comprises standing up from a chair and sitting down again, which will be repeated three times. For the current study, the performance rating was adapted to match the rating scale of the BPS, i.e., performance of mobility-related activity is rated from 0 to 3 (see Additional File 1).

#### Back pain simulation.

To induce lower back pain, a back pain simulator from Produkt + Projekt Wolfgang Moll (Niederstotzingen, Germany) is employed [68]. The back pain simulator is a standalone component of an age simulation suit, the GERonTologic simulator (GERT). It consists of a black vest resembling a motorcycle back protector (see S3 Fig), that uses six large spike rivets (three on each side next to the spine) to simulate mechanical “stabbing” pain in the lower back. The vest is secured with two shoulder straps and a Velcro waist belt that allows adjustment of its tightness, and, consequently, pain intensity. To our knowledge, this back pain simulator is the only mechanical device designed to replicate lower back pain. Initial results indicate that wearing the age simulation suit leads to a decrease in functional and physical performance of young and older adults across various tasks requiring both gross and fine motor skills [69,70]. A preliminary test with the back pain simulator on a small sample revealed similar behavioral adaptations during the BPS movement tasks. Participants tended to stiffen and straighten their backs while moving and often bent the knees rather than their backs to pick up objects.

#### Motion capture technologies.

All movements tasks are recorded with an IMU-based motion capture suit (Xsens MVN, Movella, Henderson, USA). It consists of 17 sensor nodes attached to the body with Velcro straps, recording 6D IMU data (3D acceleration and 3D angular velocity) of the whole body with a sampling rate of 60 Hz. It is widely used for motion capture in research due to its portability and reliable kinematic data output [71,72]. In previous work, it has also been successfully employed to assess the influence of acute psychosocial stress induction on full-body movements [73,74]. After attaching the sensor nodes and obtaining relevant body measures (i.e., body height, shoulder width, arm span), the system is calibrated using a standardized procedure (see [74]). The raw IMU data is wirelessly transferred to a computer running the Xsens MVN Awinda software, which processes the data through a biomechanical model, ultimately providing time-series movement information from 23 body segments with 6 different channels and joint rotation angles from 22 joints. From these movement data, we will compute a set of features that is intended to characterize the specific movements performed during the experiment, and, especially, the effect of the back pain simulator on the movement execution.

In phase 2, movements will additionally be recorded using the holographic 6D wireless locating and motion tracking system (EmpkinS H6D-Tracking), developed within the EmpkinS Collaborative Research Center [65]. The Xsens suit will then serve as a reference system for the EmpkinS H6D-Tracking. The EmpkinS system consists of wearable radio transponders – referred to as EmpkinS Beacons – that can be localized by radar base stations. Each EmpkinS Beacon additionally includes an IMU unit to record 3D acceleration and 3D angular velocity as well as an analogue frontend with electrodes to record surface electromyography (sEMG) of the muscles under the skin area the Beacons are attached to [64].

### Outcomes

#### Acceptability and feasibility (phase 1).

The main aim of phase 1 is to assess the feasibility and acceptability of various aspects of the study setup. Regarding feasibility, phase 1 will provide insights into the best way to integrate the various study components (the back pain simulator, the tasks and the measurement techniques) to identify changes in movement execution related to back pain. The results of the interview will provide information of the acceptability of the back pain simulator, the movement tasks, and the sensor technologies.

#### Pain (phase 2).

Perceived pain severity during task execution will be assessed via a VAS after completion of each movement task. Participants will rate pain intensity (0 = *no* *pain*; 10 = *extreme* *pain*), impairment due to pain (0 = *no* *impairment*; 10 = *total* *impairment*), and emotional distress due to pain (0 = *no* *emotional* *distress*; 10 = *extreme* *emotional* *distress*). The three pain ratings will be completed in both conditions after each task, resulting in 12 ratings each.

#### Fear of movement (phase 2).

Participants will rate how fearful they assess each impending movement task following its explanation but prior to its execution using a VAS scale (0 = *no fear*; 10 = *extreme fear*), resulting in 12 ratings.

#### Mobility (phase 2).

We will examine mobility by the motion capture technologies (Xsens and EmpkinS technology) and compute a set of movement features based on data from both motion capture modalities separately to compute the effect of the back pain simulator. These features are intended to characterize changes of lumbar range of motion (LROM), lumbar flexion (LF), and movement velocity (MV), among others. LF refers to the angle between the sacrum (S1/ S2) and lumbar-thoracic (L1/ T12) vertebrae. LROM refers to LF and extension, lateral bending and rotation between the above-mentioned vertebrae (S4 Fig). MV will be defined as the time participants need to execute a movement task, such as achieving maximum bending in the lifting and pick-up test.

#### Observer ratings of movement execution (phase 2).

The participants’ performance of movement execution for the six movement tasks will be rated by a trained observer in both conditions as described in Additional file 1. The scale ranges from 0 to 3 with lower ratings showing a better performance.

#### Stress (phase 2).

Task-related stress is assessed via self-report before and after each set of movement tasks. For this purpose the SSSQ-G [60] will be used, a 24-item questionnaire answered on a 5-point Likert scale (1 = *not* *at* *all*; 5 = *extremely*). As a pre- and a post-version exist, the questionnaire allows for evaluation of changes in acute task-related stress. The SSSQ-G assesses six facets of subjective task-related stress: Distress, Worry, Confidence, Negative Affect, Motivation, and Self-evaluation. Higher scores indicate a higher subjective level of stress.

Psychophysiological stress markers include unstimulated saliva samples (Salivette®, Sarstedt, Nümbrecht, Germany) to measure salivary cortisol and alpha-amylase levels indicating HPA axis and sympathetic nervous system (re-)activity, respectively. Salivary samples will be collected at four timepoints: baseline (s0), before the first set of movements tasks (s1), directly after the two sets of movements tasks (s2), and 15 min after s2 (s3). Saliva samples will be analyzed in the laboratory of the Chair of Health Psychology at Friedrich-Alexander-Universität Erlangen-Nürnberg (Nürnberg, Germany). After the collection, samples will be stored at −20°C for a maximum duration of 12 months. If longer storage times will be necessary, samples will be stored at −80°C. A maximum of two freeze-thaw cycles will be permitted. Prior to analyzing cortisol and alpha-amylase concentrations, samples will be thawed and centrifuged at 2000g for 10 min. To determine cortisol concentrations Cortisol Saliva Luminescence Immunoassays (CLIA, IBL, Germany) and for alpha-amylase concentrations kinetic/ colorimetric enzymatic assays will be used. To account for influences of menstrual cycle hormones, progesterone and estradiol concentrations derived from unstimulated passive drool samples will be analyzed using either high-sensitive Enzyme-linked Immunosorbent Assays (ELISA) or Liquid Chromatography-mass Spectrometry (LC-MS), depending on expected concentration ranges and availability of sensitive assay kits.

We will further record a 1-channel electrocardiogram (ECG) with a wearable ECG sensor (Portabiles GmbH, Erlangen, Germany; Lead I according to Einthoven’s Triangle) during the movement tasks. From the ECG, we will extract heart rate (HR) and established heart rate variability (HRV) metrics as markers for autonomic (re)activity [75].

### Explorative analyses

All outcomes for explorative analyses will be assessed at baseline in both study phases.

#### Fear avoidance.

Fear of movement/(re)injury due to physical activity will be measured with the German version of the Tampa Scale of Kinesiophobia (TSK) for the general population (TSK-G; [51,76]). The TSK-G is a modified version of the original TSK [77] for individuals with chronic pain. The questionnaire includes 17 items that are rated on a 4-point Likert scale from 1 = *strongly disagree* to 4 = *strongly agree*, with a higher total score representing higher FA. Psychometric property show acceptable internal consistency (*⍺* = .75; [76]).

#### Avoidance Endurance.

We will assess Avoidance and Endurance with the Avoidance-Endurance Questionnaire (AEQ; [52]) which focuses on pain-related behavioral, cognitive, and affective responses to pain. It consists of 49 items rated on a 7-point Likert scale ranging from 0 = *never* to 6 = *every time*. Psychometric properties show good internal consistency (*⍺* = .84; [52]).

#### Pain catastrophizing.

Pain catastrophizing will be assessed by the German version of the Pain Catastrophizing Scale (PCS; [78]). It consists of 13 Items rated on a 5-point Likert scale from 0 to 4, with a higher score indicating greater catastrophizing. The PCS is a broadly implemented questionnaire in the context of (CP)LBP (e.g., [30]) showing good internal consistency (*⍺* = .92).

#### Coping strategies for pain.

Coping with pain and pain-related distress will be assessed with the emotional pain processing questionnaire (FESV; German: Fragebogen zur Emotionalen Schmerz-verarbeitung; [54,55]). The FESV consists of 38 items and shows satisfactory reliability (*r*_*tt*_ = .78) and internal consistency (*⍺* = .77 - *⍺* = .83; [55]).

#### Chronic stress.

To assess how stressful participants perceive their life, we will employ the German version of the Perceived Stress Scale (PSS-10; [79]). Participants rate 10 items on a 5-point Likert scale from 0 to 4 with higher scores indicating higher perceived stress. The PSS-10 has been validated in a representative German community sample that revealed good internal consistency (*⍺* = .84) and construct validity [79].

### Data collection and management

When filling out the online screening-questionnaire, participants generate their personal study code, which follows them throughout the experiment. The questionnaire data are collected online and kept in accordance with the data protection guidelines of the European Union (EU-DGP). Until the pseudonymization allocation list is deleted, participants can ask for their data to be deleted at any time upon stating their assigned code. Data collected during the study are archived for 10 years before being deleted. Contact details will be maintained separately from questionnaire data. The collected data will be marked with the individual study codes to ensure pseudonymization. The list with codes will be kept separately and is password protected. All collected data will be stored in a secured research data storage system provided by the Friedrich-Alexander-Universität Erlangen-Nürnberg.

### Statistical methods

Qualitative analysis on feasibility and acceptability in phase 1 will be carried out with MAXQDA Analytics Pro [80]. Data of phase 2 will be analyzed using a repeated-measures design. Correlations of dependent variables will determine if multivariate analyses of variance (MANOVAs) or separate analyses of variance (ANOVAs) are indicated, following common recommendations of moderate intercorrelation [81].

The first MANOVA will include the within-subjects factor *condition* (PainSim + vs. PainSim-) and dependent variables measuring task-related fear of movement, pain intensity, impairment and emotional distress. A second MANOVA will assess *mobility* (LROM, LF, MV). And a third MANOVA will examine stress (HRV, salivary cortisol, salivary amylase, subjective stress) in the same manner. Psychophysiological data (e.g., HRV-data) that deviate (significantly) from the other data (i.e., outliers) will be excluded in accordance with the established practices in the literature. The pattern of missing data is tested with MCAR-test. If the missing data is missing completely at random, we will conduct multiple imputation.

## Discussion

With the planned study, we aim to establish an experimental paradigm to investigate the association of pain, FA, actual movement patterns, and acute stress responses in healthy samples under controlled pain conditions. We will realize this by employing a back pain simulator for the first time. The results of our study will provide insights in the acceptability and feasibility of simulating lower back pain.

Since the implementation of a back pain simulator is novel, the limitation of the study consists in the lack of validation of the back pain simulator. Nonetheless, we will provide initial insights into the use of this methodological approach. First tests with ten pain-free people indicated behavioral changes similar to pain patients. Further, this approach will allow us to make future studies as comfortable as possible for pain patients, thus improving prospective adherence.

Our study has two notable implications: First, it is the first study to mechanically induce lower back pain to examine mobility impairments. Second and most importantly, it integrates the assessment of pain, actual movement patterns, FA, and related stress responses measured with novel motion capture technologies.

Results of the study will set the cornerstone for future studies to not only induce pain, but also FA itself (e.g., through instructions). Our further research will focus on establishing a paradigm to reliably distinct high and low fear avoidant movement patterns with novel motion capture technologies. In the long term, such paradigms may contribute to a deeper understanding of the interplay of psychological and physiological mechanisms not only in induced pain but also in genuine CPLBP.

## Supporting information

S1 Fig Study process. (TIF)

S2 Fig SPIRIT figure with the procedure phases and data collection time points. *Control variables and covariates comprise PSS-10, TSK-G, AEQ, FESV, and PCS. **Three VAS each for pain intensity, impairment, and emotional distress due to pain after each of the six movement tasks. ***After each of the six movement tasks participants mark the locations in which they felt the pain during movement execution in a drawing of a person from behind. ****Saliva samples to determine salivary cortisol and alpha-amylase. *****The acceptability and feasibility interview is only part of phase 1.
(TIF)

S3 Fig Set-up for pain induction and movement assessment. (A) Back pain simulator (view from behind), (B) Xsens suit (executing the lifting task) and (C) model in Xsens analysis (software).
(TIF)

S4 Fig Visualization of lumbar range of motion. (TIF)

S1 File Description of the movement tasks and rating. Description of the movement tasks and rating carried out during the lab visit.
(PDF)

S2 File Feasibility and acceptability interview. Translated items of the feasibility and acceptability Interview after the experiment at the lab.
(PDF)

## Abbreviations

ADS-L: General Depression Scale (Allgemeine Depressionsskala)
AEQ: Avoidance-Endurance Questionnaire
ANS: Autonomic nervous system
BMI: Body mass index
BPS: Back Performance Scale
CLBP: Chronic low back pain
CPLBP: Chronic primary low back pain
ECG: Electrocardiogram
EmpkinS: Empatho-Kinaesthetic Sensor Technology
ER: Endurance-responses
FA: Fear avoidance
FAR: Fear-avoidance responses
FESV: Emotional pain processing questionnaire (Fragebogen zur Emotionalen Schmerz-verarbeitung)
GERT: GERonTologic simulator
HPA: Hypothalamic-pituitary-adrenal
HR: Heart rate
HRV: Heart rate variability
ICD: International Classification of Diseases
IMU: Inertial measurement unit
LBP: Low back pain
LF: Lumbar flexion
LROM: Lumbar range of motion
MV: Movement velocity
PainSim: Pain simulator
PCS: Pain Catastrophizing Scale
PSS-10: Perceived Stress Scale (10 item version)
sEMGt: Surface electromyography
SSSQ(-G): Short Stress State questionnaire (German Version)
STST: Sit-to-stand task
TSK(-G): Tampa Scale for Kinesiophobia (for the general population)
VAS: Visual analogue scale
WSQ: Web Screening Questionnaire for Common Mental Disorders
