# Supplementary material for: Sensor-based measurement of fear avoidance and movement patterns of healthy individuals with mechanically induced lower back pain during movement tasks: Study protocol for an experimental non-interventional study
Source: PLoS One. 2026 Sep 17;21(9):e0358129. doi: 10.1371/journal.pone.0358129 (PMC13585267; doi:10.1371/journal.pone.0358129)
Supplement: Supplementary file 1 [file pone.0358129.s005.pdf]

Number	Name	Picture	Instruction	Rating
1	Sock test	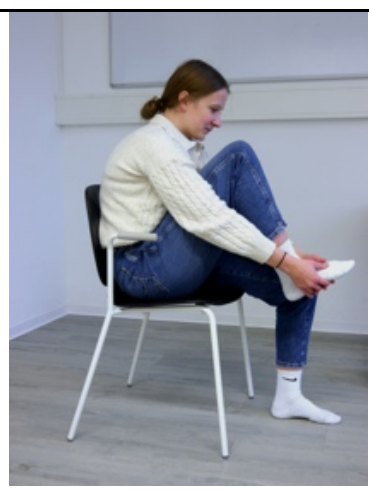	Sit down on a chair and grab toes with the fingertips of both hands when the leg is flexed in the sagittal plane. One leg will be tested at a time.	0 = Can easily grab the toes with fingertips of both hands<br>1 = Can hardly grab the toes with fingertips<br>2 = Can reach beyond the malleoli (ankle), but not reach the toes<br>3 = Hardly or not reaching malleoli
2	Roll-up test	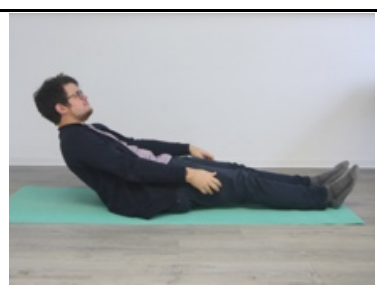	Lie down on a mat with the knees straight and the arms relaxed next to the body. Roll up slowly into a long-sitting position without using the arms.	0 = Roll up with ease<br>1 = Can roll up with marked effort or partially to long-sitting position<br>2 = Can roll up in supine between the 8th and 12th thoracic vertebra<br>3 = Roll up in supine position above the 8th thoracic vertebra
3	Fingertip-to-floor distance test	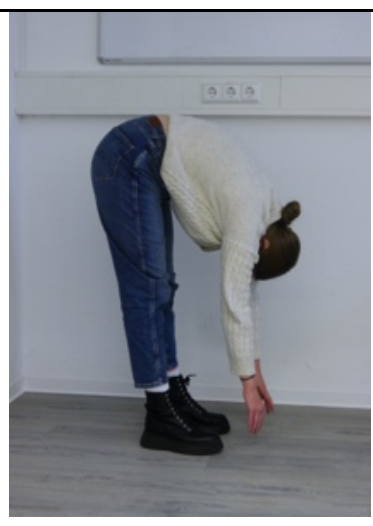	Stand on the floor with knees straight and both feet placed 10 cm apart. Reach as far as	0 = Can reach to the floor, 0 cm<br>1 = Can reach to a distance > 0 cm £ 20 cm

			possible with extended arms on the floor.	2 = Can reach to a distance > 20 cm £ 40 cm<br>3 = Can reach to a distance > 40 cm
4	Pick-up test	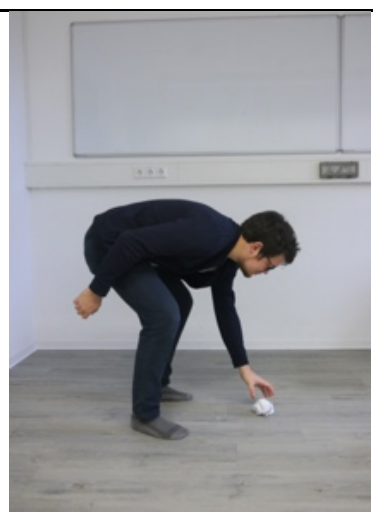	Stand upright (with a curled piece of paper lying on the floor in front of them). Pick up the curled piece of paper in three different ways to show the flexibility of your trunk	0 = Performance with ease in varied ways<br>1 = Can do the task with minor effort or some decreased variability<br>2 = Can do the task with marked effort or lack of flexibility, may need support of hand on thigh<br>3 = Cannot perform
5	Lifting test	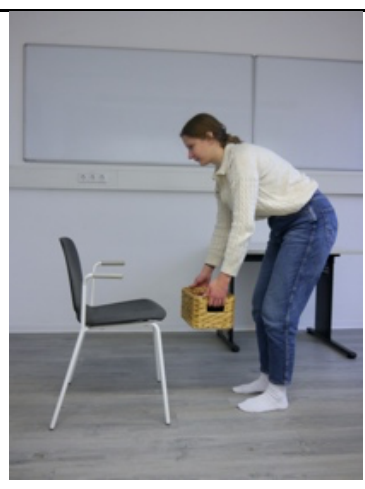	Stand upright and lift a 5 kg box from the floor to a chair and back to the floor as often as possible. Use a lifting technique of your choice.	0 = Can do > 15 repetitions<br>1 = Can do > 10 ≤ 15 repetitions<br>2 = Can do > 0 ≤ 10 repetitions<br>3 = Cannot do the task, 0 repetitions
6	Sit-to-stand-task	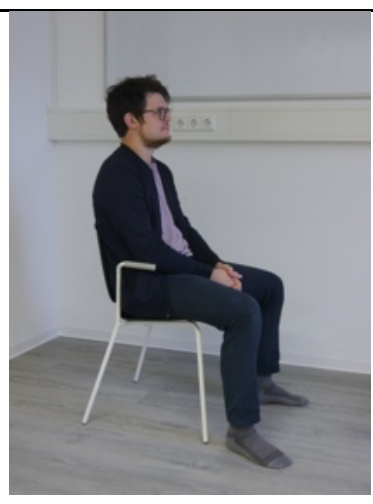	Sit on the edge of a chair. Stand up without the help of your hands and straighten your hips and knees. Sit back down on the edge	0 = Can perform the task with ease and without assistance<br>1 = Can perform the task by using their hands on their thighs

			of the chair in a controlled manner. Do this three times.	2 = Can perform the task by using the armrests for support<br>3 = Cannot perform the task or needs help
