# Supplementary material for: Sensor-based measurement of fear avoidance and movement patterns of healthy individuals with mechanically induced lower back pain during movement tasks: Study protocol for an experimental non-interventional study
Source: PLoS One. 2026 Sep 17;21(9):e0358129. doi: 10.1371/journal.pone.0358129 (PMC13585267; doi:10.1371/journal.pone.0358129)
Supplement: Supplementary file 2 [file pone.0358129.s006.pdf]

Interview after the experiment

Thank you very much for participating in this challenging study. You helped us a lot. It wasn't always that pleasant either…

(Wait for spontaneous comments; if necessary, ask further about how it was for them, what was particularly unpleasant…

____

____*)*

Now I would like to ask you how you perceived different aspects of the experiment. Your answers are, of course, voluntary, so you are not obliged to answer, and you may stop the conversation at any time or skip questions.

1. Back Pain Simulator

Ultimately, our aim is to investigate individuals suffering from back pain. That's why we used the vest intended to simulate back pain.

How did it feel to wear the back pain simulator?

____

Could you describe that in a little more detail?

____

What thoughts went through your mind while wearing it?

____

Did you move differently because of the back pain simulator?

____

Did you use any strategies to handle the back pain simulator / the pain?
(If no answer: e.g., Did you try to keep your back as straight as possible?)

____

____

Did you at any point feel like discontinuing because it was too uncomfortable?

____

What would you have thought if you had seen the spikes of the back pain simulator before the experiment and explicitly had been told about them?

____

____

What do you think about possible consequences of wearing the back pain simulator?
(If fear/worry: emphasize again that no permanent damage can occur since it is a certified device.)

____

____

Have you ever had pain in your **lower** back? ________________
Could you describe this in more detail?

a. *If yes:*
    i. What type of back pain? ______________________________
    ii. What was the cause of the back pain?
    ____
    iii. How long did the back pain last? _______________
    iv. Is this pain comparable to the back pain simulator? *(Ask about similarities and differences.)*
    ____
    ____
b. *If no:*
    Do you think real lower back pain would feel like it did with the back pain simulator?
    ____

2. Exercises

Earlier, you performed six exercises.

How was that for you?

____

Is it okay if we go through the exercises one by one again, and you describe how each one was/felt for you?

1

2

3

4

5

6

(Remind briefly what each exercise was and note answers in the performance log with rating and observations. (If appropriated: Ask follow-up questions based on previously noted observations.))

What was the most difficult for you?

____

What was the easiest for you?

____

At which point was the difference between the two runs the greatest?

____

And where the smallest?

____

(If participant was unable to perform one or more exercises:

I had the impression this exercise wasn't easy for you.

Could you describe what made it difficult?

Ask in detail whether it was due to the back pain simulator, pain, or something else.)

____

____

(If necessary, validate that the exercises are difficult, especially with the suit on. "We also tried all the exercises ourselves and didn't all manage them equally well.")

3. <u>Measurement Sensors</u>

We attached quite a lot of measurement sensors to you overall (that was the Xsens suit).

How was that for you?

____

Did anything bother you a lot?

____

Would you say it bothered you so much that it made the movements even harder?

(If yes: What would need to be different to make it better?)

4. **Final Questions**

Would you participate in the experiment again?

(If participant discontinued the experiment:)

What made you discontinue the experiment?

What would have needed to happen for you to continue the experiment?

Is there anything important that we haven't covered yet but that you would like to share with us? (Do you have further comments on the experiment as a whole?)

Thank you for taking the time for this interview. This is very helpful for us because it allows us to further develop the experiment. Thank you very much! You're done now!
